# Regulation of choroid plexus development and its functions

**DOI:** 10.1007/s00018-022-04314-1

**Published:** 2022-05-19

**Authors:** Petra Kompaníková, Vítězslav Bryja

**Affiliations:** 1 Department of Experimental Biology, Faculty of Science, Masaryk University, 62500 Brno, Czech Republic; 2 Department of Cytokinetics, Institute of Biophysics, Academy of Sciences of the Czech Republic, 61265 Brno, Czech Republic

**Keywords:** Choroid plexus (ChP), ChP epithelial cells, Morphogenesis, Cerebrospinal fluid (CSF), Cortical hem, Rhombic lips

## Abstract

The choroid plexus (ChP) is an extensively vascularized tissue that protrudes into the brain ventricular system of all vertebrates. This highly specialized structure, consisting of the polarized epithelial sheet and underlying stroma, serves a spectrum of functions within the central nervous system (CNS), most notably the production of cerebrospinal fluid (CSF). The epithelial cells of the ChP have the competence to tightly modulate the biomolecule composition of CSF, which acts as a milieu functionally connecting ChP with other brain structures. This review aims to eloquently summarize the current knowledge about the development of ChP. We describe the mechanisms that control its early specification from roof plate followed by the formation of proliferative regions—cortical hem and rhombic lips—feeding later development of ChP. Next, we summarized the current knowledge on the maturation of ChP and mechanisms that control its morphological and cellular diversity. Furthermore, we attempted to review the currently available battery of molecular markers and mouse strains available for the research of ChP, and identified some technological shortcomings that must be overcome to accelerate the ChP research field. Overall, the central principle of this review is to highlight ChP as an intriguing and surprisingly poorly known structure that is vital for the development and function of the whole CNS. We believe that our summary will increase the interest in further studies of ChP that aim to describe the molecular and cellular principles guiding the development and function of this tissue.

## Introduction

**Fig. 1:**
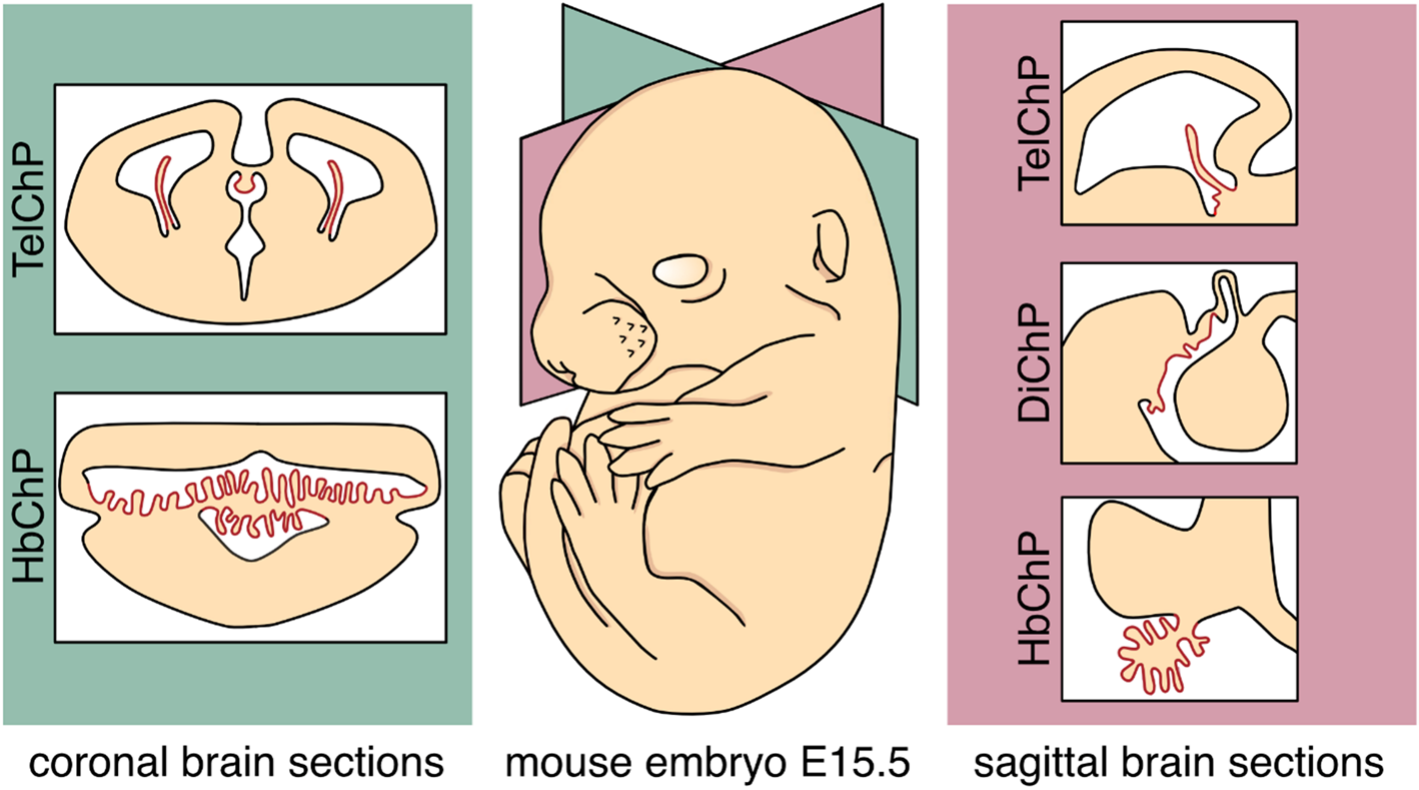
The localization of the ChP secretory system within the developing mouse brain. The set of three distinct ChP types (highlighted in red colour) occupies every ventricle formed within the main brain embryonic regions. This includes lateral ventricles marked by the presence of telencephalic TelChPs, 3rd ventricle characterized by the small DiChP and 4th ventricle fills with the robust HbChP. A growing body of evidence indicated differences between the individual types of ChP, not only in their diverse morphological appearance, which can be appreciated on the coronal (panel in dark green) as well as sagittal sections (panel in dark pink) of the murine brain, but also in the expression profiles of the ChP epithelia. This spatial regionalization translates into differences in the composition of the cerebrospinal fluid (CSF) which ChPs produces into the lumen of the ventricular system. *ChP* choroid plexus, *TelChP* telencephalic choroid plexus, *DiChP* diencephalic choroid plexus, *HbChP* hindbrain choroid plexus, *CSF* cerebrospinal fluid (CSF), *E* embryonic day

The choroid plexus (ChP) is a conspicuous tissue within the brain ventricular system of all chordates about the lancelets [1] which is widely recognized as the predominant source of the cerebrospinal fluid (CSF) [2]. This greatly specialized structure protrudes into brain cavities from three distinct brain regions: the pair of Telencephalic ChPs (TelChP), also known as lateral ChP, extends symmetrically into lateral ventricles of the telencephalon, (2) Diencephalic ChP (DiChP), also known as 3 ChP, lays in the 3rd vesicle formed in the diencephalon, and finally, (3) Hindbrain ChP (HbChP), also known as 4th ChP, resides in the 4th cavity within the mesencephalic region. (Fig. 1) Regardless of ChP first observation by the Greek anatomist Herophilos already before our era [3], severe pathologies of this tissue reported at the beginning of the last century [4, 5] and its fairly well-defined structure available from the 1960s [6, 7] the growing interest in ChP among neuroscientists is evident only in the recent decade. Several facts lie at the root of this shift in the perception of ChP tissue and its significance. Besides model and methods availability or sufficiently demonstrated clinical salience, the disclosure of the ChP functional potential to actively secrete morphogens into the CSF and thus participate in the patterning of the adjacent neuronal tissue across the neural tube took the essential lead [8–10].

This review aspirates to compile the current knowledge about the ChP with an emphasis on its development. Within this manuscript, we attempted to bring a new synthetic view on the most crucial steps of the ChP development and to comprehensively summarize known functions of molecular regulators involved in the ChP ontogenesis. We distinguished and schematically outlined three key processes of ChP development and comprehensively described the key factors and molecules needed for the specification and formation of this tissue. In addition, our review also provides the reader with clearly summarized essential information about the ChP morphology assessment, the experimental models to study the development of ChP, the commonly used molecular markers, and the currently available specific mouse strains used for studies of ChP. We believe that this summary will provide a comprehensive insight into ChP development and morphogenesis, and will stimulate and efficiently direct further research toward our understanding of this crucial secretory tissue.

## ChP morphology

ChP strikes the eyes of the neuroscientists certainly by their gross morphological shape in the lumen of ventricles. Within the central nervous system (CNS), this tissue represents rather atypical structures with huge differences in the overall appearance of individual ChPs (Fig. 1). In the murine brain, the HbChP displays the most complex three-dimensional structure with numerous branches bifurcating in the hindbrain cavity whose length and a total number have become evaluation parameters of the HbChP morphology [10]. Moving more anteriorly across the neural tube, the morphology assessment of the frond-like DiChP is still quite challenging and sporadically realized as this miniature structure has less-defined borders [11]. Recently, Langford and colleagues approached this issue by the manual track of Transthyretin + epithelial cells of ChP (ChPEC) on the histological sections with the intention to determine the length of the DiChP [12]. Finally, the tarpaulin-like tissue of TelChPs occupies nearly the entire telencephalic cavities with an exception of their frontal horns [13]. The morphology of this ChP type has been scored as an evaluation of its total area and length within the lumen of the ventricles [10, 12].

**Fig. 2:**
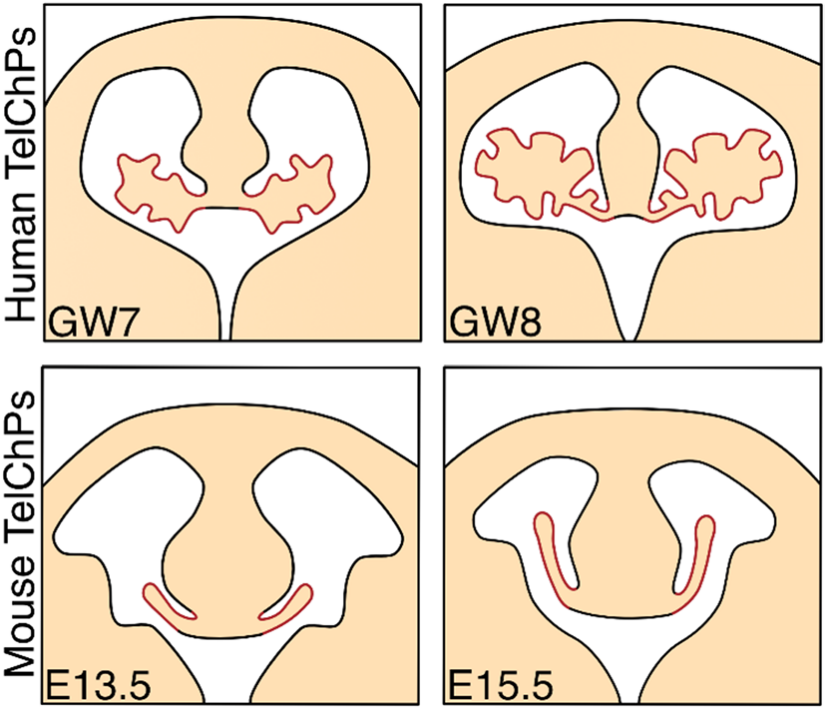
The distinct morphological shapes of TelChPs in mouse and human. The TelChPs outgrows gradually out of the medial telencephalic wall into the lateral ventricles. TelChPs fill up to 63% of the total cavity area in humans [16] while the murine TelChPs occupied only 27% of the mapped area [17]. These differences are due to a more complicated morphology of human TelChPs. *TelChP* telencephalic choroid plexus, *GW* gestation week, *E* embryonic day

Further divergences in the ChP morphology can be found across the individual species, namely between DiChPs and TelChPs of various animals [14]. The striking differences in the morphological appearance have been observed in the human TelChP compared to its murine form. TelChPs within the human brain display a more corrugated character of the tissue resembling the branched morphology of the HbChP (Fig. 2) [15]. However, the molecular mechanism which stands behind this morphological alternation has not been clarified and additional research is needed to determine the regulatory network which participates not only in this phenomenon but also in the general morphological specification of ChP across the ventricles as well as species.

Overall, quantitative analysis of ChP morphology is not trivial. It can be performed by measurements on several consecutive slides of the brain sections, which, however, still does not provide an overall view of ChP within the brain ventricular system. To overcome this obstacle, Perin et al*.* executed the three-dimensional reconstruction of the rat HbChP tissue using the light sheet microscope scans focusing on its total macrostructure as well as on the individual components such as vasculature [18]. Further information can provide an evaluation of the relative position of ChP within the ventricle. For example, the position of HbChP within the 4th ventricle has been used as a differential criterion in the diagnosis of the human posterior fossa malformations like Vermian hypoplasia [19] or Blake's pouch cyst [20].

## ChP structure and cellular composition

The gross structure of ChP, shortly described below, is conserved across the aforementioned ChP types, as well as species. The ChP tissue is constituted out of two core components—the stromal tissue mass which is enveloped by the single monolayer of polarized cuboidal epithelial cells [21].

Mesodermally derived stroma [22] is composed of a plethora of cell types, which involve fibroblasts, glial, immune, neuronal, or endothelial cells [11]. The last-mentioned cells form an atypical vascular system, which is unique to ChP and has the diaphragmed fenestrations in capillary walls. These modulations ensure the influx of water and small molecules from the blood to epithelial cells which are elemental for the CSF secretion [23, 24]. The formation and effective function of the ChP vascular network is guaranteed by the presence of the smooth muscle cells together with the pericytes that cover endothelial cells [11].

ChP vasculature is connected to the brain circulation system. LatChPs are supplied by the anterior as well as posterior choroidal arteries which are a continuance of the internal carotid and the vertebral artery, respectively. Similarly, the vertebral artery also delivers the necessary blood to the DiChP. Within the hindbrain region, the basilar and vertebral arteries branched to the anterior and posterior inferior cerebellar arteries which outgrow to the whole HbChP [25, 26].

In addition, a broad spectrum of the immune cell types, including monocytes, lymphocytes, basophils, neutrophils, dendritic cells or B cells can be found in the ChP stroma. However, the most abundant immune cell population are macrophages [11] which are derived from the haematopoietic stem cell-derived myeloid cells that are produced within the aorta-gonad-mesonephros region or later in the development by the fetal liver [27]. Interestingly, the apical surface of choroid plexus epithelial cells (ChPEC) is occupied by the specific subpopulation of macrophages termed as epiplexus or Kolmer cells, which scan the content of CSF [28].

The second-mentioned element of the ChP tissue—the ChP epithelium—originates in the neuroectoderm and represents the continuance of the ependymal lining of the ventricular system [29]. The population of ChPEC is distinguishable from others by three major features which are the results of the ChPEC maturation and reflect functions of this secretory tissue. First, the space between ChPEC is sealed by tight junctions which restrain the paracellular transport across the ChPEC and establish the physical barrier between blood and ventricles fulfilled by CSF [30]. Second, the composition and surface of epithelial cells are significantly adjusted to produce CSF. Namely, the epithelial sheet displays higher mitochondrial content [31] together with asymmetrical localisation of various transporters on the apical (e.g., Na^+^- K^+^- ATPase or water channels) as well as a basal (e.g., Na^+^- HCO_3_^−^ exchanger or anion exchanger 2) side [32–35]. Third, the apical side of the epithelium is characterized by the presence of membrane modulations such as microvilli or cilia which significantly increase the area out of which the CSF is produced [7]. The most typical molecular markers used to distinguish individual cellular pools of developing ChP are summarized in Table 1.

**Table 1:** List of molecular markers most commonly used to study the development of choroid plexus

Marker	Biological function/note	Cell type	Method	References
Ttr	Transthyretine; hormone transporter	ChPEC	ISH, IF	[12, 37–41]
Aqp1	Aquaporin 1; water channel essential for the CSF production	ChPEC	IF	[10, 39, 42]
Cla1	Claudin 1; tight junction composition and integrity of	ChPEC	IF	[40, 43]
Foxj1	Forkhead box protein J1; Transcriptional factors required for the formation of cilia	ChPEC	ISH	[40]
Zo-1	Tight junction protein ZO-1; Tight junction composition and integrity	ChPEC	IF	[12]
Otx2	Orthodenticle homeobox 2; homeobox transcription factor essential for brain development	ChPEC	IF	[44]
Slc4a10	Sodium-driven chloride bicarbonate exchanger; protein which plays an important role in regulating intracellular pH	ChPEC	IF	[45]
ColIV	Collagen alpha-4 (IV) chain; structural component of basement membrane	The lamina densa	IF	[37]
Pecam1	Platelet endothelial cell adhesion molecule; cell adhesion molecule activating leukocyte transendothelial migration	Endothelial cells	IF	[37, 44]
Cspg4, Ng2	Chondroitin sulfate proteoglycan 4, Proteoglycan AN2, Proteoglycan stimulating proliferation and cell migration	Pericyte	IF	[44]
Desmin	Muscle-specific type III intermediate filament essential for proper muscular structure and function	Pericyte	IF	[37]

Despite shared basics, ventricle- or age-dependent variations of individual cell types has been shown by recent single-cell analysis of all ChPs across the mouse lifespan. Interestingly, this study uncovered further regionalization within the ChPEC. These spatial divergences were most typical for the epithelium of the HbChP which could be further subdivided into the rostral (*Penk*^+^*, Shh*^+^*, Wnt5a*^+^ among others) and the caudal (i.e., *Fab3*^+^*, Acad8*^+^*, Rb1*^+^*)* parts [11]. Similarly, despite the molecular details being missing, even the very early study in humans morphologically distinguished anterior and posterior regions within the epithelium of TelChP [36].

## CSF fluid

The ChP is a superior secretory tissue that supplies the brain ventricular system with the CSF, producing up to 80% of its total volume [46]. The human ventricular system is filled with approximately 150 ml of CSF and this volume is daily changed 3–4x [47]. There is a general agreement about the CSF flow. Its directional movement starts from lateral ventricles to the 3rd and 4th cavity through the foramen of Monro and cerebral aqueduct, respectively. Afterward, CSF enters the subarachnoid space and spinal cord via the foramen of Magendie, where it is resorbed back to the bloodstream. On top of that, several studies suggested novel ways of CSF absorption highlighting cervical lymphatics or dural venous plexus [48]. The CSF flow is guaranteed by the synchronic cilia beating of the ependymal lining which is tightly regulated by the circadian rhythm [49] together with brain-derived hormones [50]. Within the zebrafish ventricular system, the motile ependymal cilia appear to regulate the CSF flow merely within the induvial ventricles, while the CSF motion across cavities is generated by the heartbeats along with the body movements [51].

Formerly, CSF was mainly recognized as a clear body liquid serving a mechanistic function within the CNS—ensuring its protection, cleanliness, as well as preservation of intracranial pressure needed for the ventricle expansion and proper brain development [52]. Nowadays, CSF is perceived as a dynamic fluid with a heterogeneous morphogen composition that arises from the divergent secretion activity of each ChP epithelia, which is further described in the chapter *ChP-mediated brain patterning*. Interestingly, this phenomenon seems to decrease over the lifespan with its peak during embryonic development [53, 54]. Thus, CSF regionalization contributes significantly to the diverse temporal developmental programs of stem cells pools that reside in the interface of individual ventricles [8, 55].

Another noteworthy fact about CSF is its brain nourishing character as this body liquid contains several micronutrients, ions, peptides, or a variety of small RNAs [21, 56]. Besides this, the macronutrients like glucose or lactate pass through the fenestrated capillaries and any imbalance in their concentration levels within the CSF can signify some pathological conditions. For example, decreased CSF level of glucose has been connected to bacterial meningitis [57] or leptomeningeal carcinomatosis [58]. Lower lactate parameters within the CSF have been observed in patients with cerebral hypoxia [59] or neurometabolic disorder [60]. On top of this, ChP tissue tightly coordinates the penetration of the leptin from the blood into the CSF fluid. This circulating hormone stimulates receptors of the hypothalamus which culminates in the control of satiety feeling [61].

## Development of the ChP

**Fig. 3:**
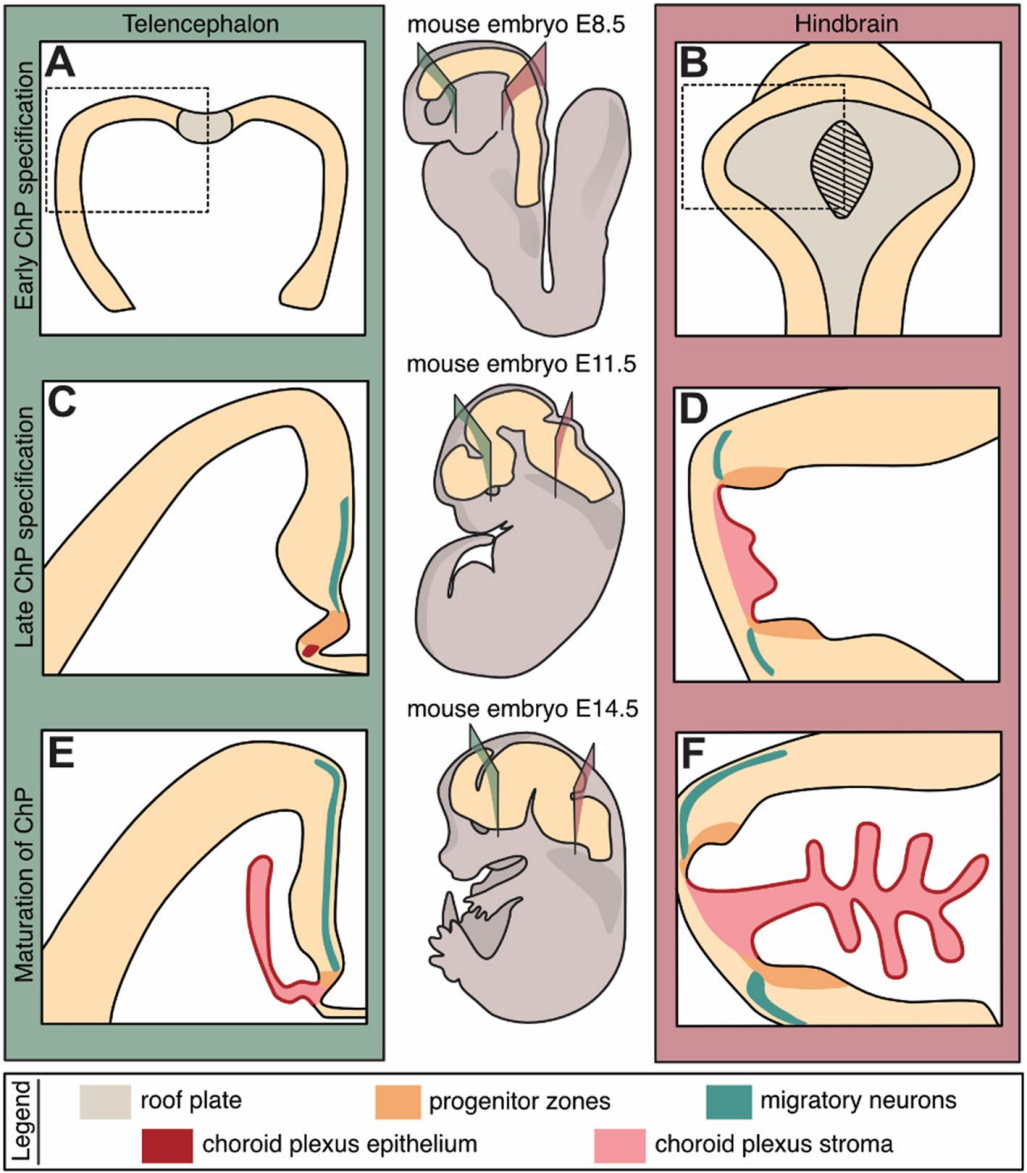
The ontogenesis of ChPs. A schematic simplification of ChP development and formation is shown on the coronal brain sections of mouse telencephalon (panels in green, left) and hindbrain (panels in dark pink, right) at E8.5 (**A**, **B**), E11.5 (**C**, **D**) and E14.5 (**E**, **F**). The early specification of the TelChP as well as HbChP epithelial cells (ChPEC) (highlighted in red within panels **C**–**F**), takes place in the embryonic roof plate (in grey; except the hatched part in **B**) (**A**, **B**). Further development (panels C,D as close up of neural tube regions indicated by dashed boxes in **A**, **B**) of the ChPs is connected with the formation of progenitor domains (orange). They are called cortical hem (CH) in the telencephalon (C) and rhombic lips (RL) in the hindbrain (D). These regions, besides ChPEC, also give rise to the migratory neurons (green). They are represented by Cajal–Retzius cells (from CH) migrating into the developing cortex of the telencephalon (**C**, **E**) as well as hindbrain migratory neurons targeting the cerebellum (from upper RL) (**D**, **F**). The last step in the ChP ontogenesis is its maturation when ChP gets its typical shape (**E**, F). Maturation involves further differentiation of ChPEC and the formation of ChP stroma (pink) achieved by the migration of mesoderm-derived stromal cells

**Fig. 4:**
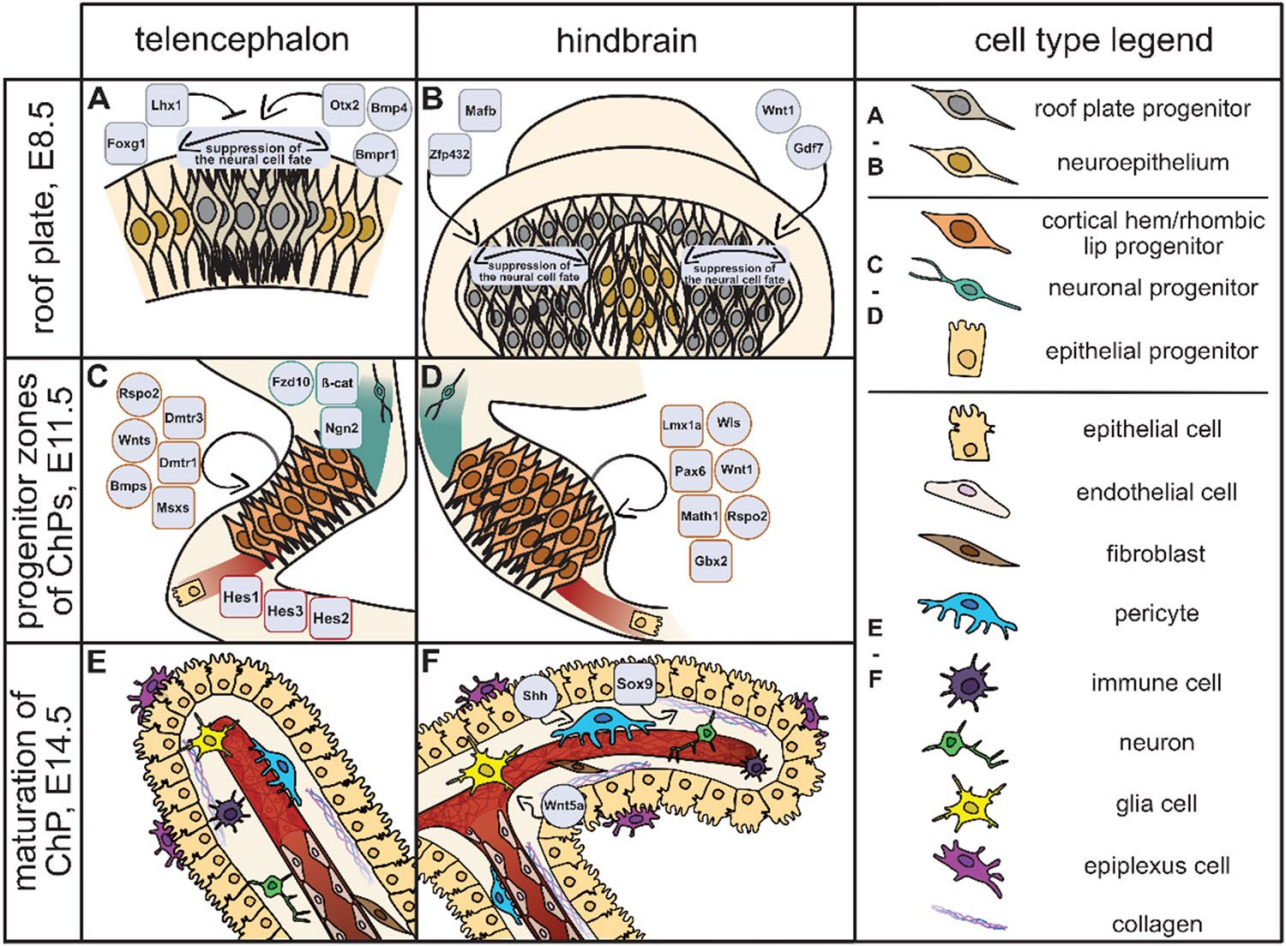
Cell types, transcription factors, and signalling pathways involved in the ontogenesis of ChPs. A summary of cell types and key molecular regulators involved in the development of ChP. Schematics directly connects to Fig. 3 that describes the roof plate (**A**, **B**; E8.5), progenitor zone (**C**, **D**; E11.5), and maturation (**E**, **F**; E14.5) stages of ChP development. The development of TelChP (**A**, **C**, **E**; left) and HbChP (**B**, **D**, **F**; right) is outlined separately. For the legend of individual cell types, see the panel on the right. Transcription factors are shown in rounded squares; components of morphogenetic signalling pathways are in circles. For further details and references, see the text in the chapter “Development of the ChP”

The ChP ontogenesis is a complex and dynamic process that consists of several crucial consecutive steps leading to the formation of the fully-functioning ChP tissue. These stages will be individually discussed in the sections below and are schematized in Fig. 3 and further expanded to the cellular and molecular level in Fig. 4. Of note, ChP development is predominantly studied in the mouse. However, we would like to appreciate the fact that several noteworthy studies, not further discussed in this review, have been done in other models such as zebrafish (for primary references see [62–65]). Additional imbalance in the knowledge on ChP development can be found in the levels of published details about the formation of individual ChP types. Namely, the DiChP is repeatedly omitted from the analysis probably due to its unclear morphology. On top of this, the vast majority of the studies focused on the development of ChP were performed on the systemic knock-out models which impoverish our knowledge about the time and cell-type requirements for the specific proteins within the individual developing stages of ChP. Only more recently tissue- and/or time-specific ChP knockout models were reported. To help the reader, we summarize the mouse strains that are most commonly used to specifically study ChP development in Table 2.

**Table 2:** The mouse strains used to specifically target the ChP tissue within the developing neural tube

Strain	Targeted cell type	Note	References
*Foxj1*-Cre^ERT2^	Ciliated ChPEC	Conditional	[10, 40]
*Pax2*-Cre	HbChPEC	Effected the rostal part of HbChPEC	[113]
*Wnt1*-Cre2	Roof plate progenitors		[40]
*Wnt1*-Cre	Roof plate progenitors	Have been observed to cause developmental phenotypes by ectopic activation of Wnt signaling [114]	[55]
*Gdf7*-Cre	Roof plate progenitors		[55, 38]
*Otx2*-Cre^ERT2^	Dorsal midline cells	Conditional	[38, 44]
*Gli1*-Cre^ERT2^	Rhombic lip progenitors	Conditional	[55]
*Tie2*-Cre	Endothelial cells		[44]
Vegf-lacZ	Endothelial cells		[44]

## Early ChP specifications

The first sights of ChP tissue within the murine brain have been observed at embryonic day (E) 11.5 when the HbChP start to bulge into the 4th ventricle. Shortly after, TelChPs arise from the medial wall of the telencephalon quickly followed by the DiChP formation within the 3rd cavity [7]. Nonetheless, the specification of the ChP occurs earlier in the development as indicated by grafting experiments of future chick ChP which was capable to form ChP tissue out of its original region [66]. This concept was further extended by the in vitro cultivation of the E8.5 and E9.5 mouse prospective ChP regions from relevant brain regions which also resulted in the formation of ChP like structures [29].

ChPs are adjacent to the roof plate which is a fundamental signaling hub that occupies the dorsal midline of the developing CNS [67]. Roof plate cells, which are located ventrally to the prospective neural crest cells within the neural tube [68], settle the dorsal midline region after the emigration of their neighbors [69]. Several studies have focused on this region to resolve the potential role of these apicobasal polarized cells in the ChP specification.

The lineage tracing experiment of *Gdf7* + cells occupying the dorsal midline uncovered the contribution of these cells to the epithelium of HbChP as well as DiChP from E9.5. The examination of the TelChP showed the presence of a small *Gdf7* + cellular population within the anterior part of the TelChP epithelial sheet. Contrarily, the posterior part of the epithelium lacked any progeny of *Gdf7* + cells labeling [70] which suggests the existence of regionalization of the mouse TelChP epithelium as described previously in humans [36]. A further study addressing the involvement of roof plate in the formation of HbChP epithelium divided the hindbrain roof plate into three spatiotemporal regions which emerge from the neuroepithelium at distinct time points. Field 1, positive for *Wnt1*, colonized the dorsal midline at E8-E9.5 and did not participate in the formation of the ChP epithelium. On the other side, fields 2 and 3 positive for *Wnt1* and *Gdf7* formed at latter E9.5 from rhombomeres 2–8 and rhombomeres 1, respectively, clearly contributed to the epithelium of HbChP [71]. These observations of Wnt1 significance in the development of HbChP can be further complemented by the phenotype of *Wnt1* knock-out mice that lack this tissue [72].

In general, the ChPEC specification requires inhibition of the neural fate within the neuroectoderm from which these cells arise [29]. The regulatory network behind this process has been studied widely in the embryonic telencephalon. For instance, members of the BMP protein family, which are present within the dorsal parts of the telencephalon from E10.5, induce epithelial cell fate within the dorsal midline by the repression of the Lhx1/Foxg1 complex [37, 73–76]. This observation was validated experimentally in vitro by Watanabe and colleagues who showed that Bmp4 is sufficient to induce ChPEC fate in neural progenitors derived from mouse embryonic stem cells [77]. Furthermore, the misexpression of Bmp receptor *Bmpr1* at E10.5 resulted in the expansion of ChPEC, whereas its deletion decreased the number of ChPEC [78]. This specification process is also regulated by the Otx2-Emx2 transcriptional network which is present in this region from E10.5 [79]. The ectopic expression of *Emx2* within the dorsal midline has been observed to activate the neuronal cell fate trajectories. The absence of ChPEC in these mice that was accompanied by the decreased levels of *Otx2* transcripts directed further research of pro-epithelial factors within the roof plate to Otx2 [80, 81]. Indeed, the early deletion of the *Otx2* gene at E9.5 caused the absence of all ChPs, while its later ablation (at E15.5) impaired only the development of the HbChP [38]. Altogether, these studies point to the crucial role of Otx2 in diverse time points of the ChP development.

## Text Box: How to study ChP and its development

**Table:** 

*Molecular markers* To distinguish ChP from the surrounding tissue, several representative markers can be used to visualize the ChP epithelium. For instance, the transporter of thyroid hormone thyroxine T4 throughout the CSF—Transthyretin (Ttr)—is exclusively present in ChPEC from the early stages of ChP development, even before the ChP starts to protrude into ventricles, and its expression persists till adulthood [82]. The differentiated epithelial sheet can be also highlighted by the water channel Aqp1 [10] or other proteins essential for the ChP secretory functions like Slc4a10 [45]. The transition zone between the progenitor and fully differentiated epithelial cells can be visualized by ciliogenesis markers like *Shisa8*, *Ccdc67* or *Mcidas* [11]. For summary overview see Table 1. *Mouse models* Several tissue-specific mouse models have been developed to study ChP development. A strong emphasis must be placed on the selection of the adequate Cre drivers, as the transcriptomic regionalization of ChP epithelium can significantly influence the outcome of the analysis. The potential disruption of the ChP morphology arising in these models has been evaluated by the manual measurements of several parameters (e.i. total area, length or number of branches [10, 40] on several consecutive tissue slides. In the future, these interminable and challenging quantifications should be replaced by the automatized procedure capable to assess the ChP tissue properties in three dimensions, as lately was shown by Perin and colleagues [18]. For detailed overview of the mouse models see Table 2. *ChP organoids* The recent generation of the human ChP organoids has uncovered several possible directions of the ChP research, in the terms of the result validation of big-data analyses as well as exploration of ChP functions in vitro or evolutionary studies of ChP development. These TelChP-like protrusions on the cerebelar organoids derived from human pluripotent stem cells displays a high level of similarities to those in vivo regarding the secretome or ChPEC transcriptomic profile. Nonetheless, this state-of-art model lacks the vasculature network within the stroma and as such it is unsuited for the interaction studies of endothelial cells with other cell types within the ChP [41]	

Moving more posteriorly across the neural tube, the recent investigation of the zinc-finger Zfp423, expressed within the hindbrain roof plate from E8.5, revealed its strong pro-epithelial functions. The C-terminal mutants of this transcriptional factor display hypoplastic HbChP and the downregulation of another key player in the HbChPEC specification—*Lmx1a* [83, 84]. Mutants of this transcriptional factor are characteristic of drastic malformations within the hindbrain region, including an underdeveloped roof plate that culminates in the absence of HbChP [84, 85]. One of the transcriptional targets of Lmx1a is Mafb. The analysis of *Mafb*-deficient mice from E10.5 showed delayed differentiation of HbChP probably caused by increased apoptosis together with the decrease of the proliferation within this tissue. Interestingly, *Mafb* mutant tissue had lower levels of Lmx1a [39] which suggested the complex regulatory network required for the specification of HbChPEC within the hindbrain roof plate. Further effort is required to fully comprehend the early specification of HbChPEC.

## Progenitor zones of the developing ChP epithelial cells

Later, development (E11.5–E14) of TelChPEC and HbChPEC is linked with two highly proliferative domains that are contiguous to the developing ChP—telencephalic cortical hem and rhombic lip residing within the hindbrain region. These structures represent not only the progenitor zones of ChPEC but also the origins of migratory neurons.

The cortical hem is a dorsal midline derivative whose development is regulated by the balance between functions of Shh signaling [86, 87] and LIM-homeodomain transcription factor Lhx2 [88]. This Wnt/Bmp/Msx-rich region [87] is apart from TelChPEC, one out of three sources of Cajal–Retzius cells that migrate tangentially to the cortex [89] or hippocampal marginal zone [90]. The rhombic lip is a territory placed along the dorsal midline which is further divided into two regions—upper rhombic lip (URL) and lower rhombic lip (LRL) [91]. Apart from the epithelial cells of ChP, the Wls/Math1/Pax6-positive URL [92] gives rise also to tangentially migrating granule progenitor cells of the cerebellum [93], whereas migratory mossy fiber neurons or climbing fiber neurons have been observed to arise from the LRL [94].

A recent study connected these two progenitor domains by a shared expression profile typical of high expression of *Rspo* genes [11]. *Rspo* encodes the members of the R-spondin family which act as secreted enhancers of the Wnt/ß-catenin signaling pathways [95]. Moreover, the transcriptomic analysis of all embryonic ChPs identified *Rspo2* as a common marker of progenitor cells which, based on the in silico lineage trajectory analysis, can further produce all relevant neurons and epithelia of ChPs [11]. Nonetheless, the regulators that control the balance between neuronal and epithelial differentiation of these progenitors are largely unknown, with few exceptions.

In the cortical hem, the interplay between the Hes-1,-2, and -3 transcriptional factors and Neurogenin2 (Ngn2) contributes to the equilibrium between ChPEC and Cajal–Retzius pools. The *Hes* mutants displayed upregulation of Ngn2 expression which acts in the favor of Cajal–Retzius cells numbers, while the population of ChPEC decreased [96]. Furthermore, the lineage tracing of *Fzd10*, encoding a Wnt receptor, showed the exclusive contribution of *Fzd10* + cells to the pool of Cajal–Retzius cells [90], indicating that the quantitative balancing of the individual cell types may also occur on the receptor levels and subsequent downstream signaling cascades. Indeed, the most recent analysis of one of the main Wnt signaling branches—canonical Wnt/ß-catenin cascade—within the cortical hem revealed the necessity of its balanced level within this brain region as constitutive activation of ß-catenin enhanced neuronal cell fate trajectories within the cortical hem, to the detriment of the epithelial state [97]. Additionally to this, a recent study pointed out the vital role of double-sex and mab-3 related transcription factor (Dmrt) genes in the formation of hem-derived Cajal–Retzius cells, as the Dmtr1, Dmtr3, and Dmtr1/Dmtr3 double knockout out mice displayed a significant reduction of cortical hem region and lower number of these cells [98]. Unfortunately, the authors did not examine the effect of these transcriptional factors on the ChP epithelial pool.

The regulation cascade which fine-tunes the epithelial vs. nonepithelial pools within the URL is less described as scientists concentrated predominantly on the cerebellum. Still, several studies do exist. For instance, *Lmx1a* KO mice display several malformations within the cerebellum as well as a significant reduction of HbChP size [85]. The lineage tracing of the *Gbx2* + cell progeny showed their contribution both to the granule cell layer of the cerebellum as well as HbChP epithelium. Interestingly, contribution to granule cells persisted to adulthood, whereas contributing to the outgrowing epithelium of HbChP decreased rapidly even during the early embryonic stages [99]. On top of this, a study performed by Huang and colleagues pointed to the role of Shh morphogen in the proliferation rate of LRL progenitors which can significantly affect the size of HbChP [42]. However, other LRL derivatives were not examined in this study.

Overall, the specification of the ChPEC is a complex multistage process that involved a myriad of transcriptional factors or morphogens which ensure the cell fate switch of ChPEC from the initial neuroectodermal character. Furthermore, an additional equitable network of regulators within later progenitor domains of ChP epithelia is needed to complete the differentiation of ChPEC as well as other cell types derived from these brain regions.

## The maturation of ChP

Once the ChPEC cells are defined, the whole tissue undergoes the maturation process. Based on the morphological changes of human TelChPEC, this process has been divided into four stages [100]. Epithelial cells of Stage I are characterized by the central localization of nuclei and the absence of apical membrane folding. These cells form pseudostratified epithelium consisting of 2–3 layers. The transition to Stage II involves changes in the shape of epithelial cells to cuboidal and the shift of the nuclei toward the apical side. Additionally, the basal connective tissue starts to form. Stage III is mainly defined by the formation of the cilia on the apical side. Moreover, epithelial cells display a higher number of villi and basally located nuclei. Large modifications also occur within the stroma as the complexity of the vascular network increases gradually. The gradual decrease in the epithelial cell size defines the switch to the last 4th Stage [100].

It is worth mentioning that, under normal circumstances, epithelia of ChPs are quiescent cell populations whose cell division is dramatically reduced early after the maturation [101–103]. However, the examination of ChPs in some pathological conditions like stroke [104] or physical brain injuries [105, 106] revealed an increased proliferation rate of ChPEC which led to the speculation that the ChP is a depository of neural progenitor cells in the adult mammalian brain [104]. Interestingly, grafting experiments of HbChP cells into spinal cord lesions significantly enhanced both the tissue repair and overall locomotor movements of the animals suffering from spinal cord injury [107, 108]. Similarly, courses of stroke [109], hydrocephalus [110], or Huntington's disease [111] have been significantly improved after the transplantation of mature ChPEC into the rodent models of these diseases. This highlights the ChP as tissue with promising therapeutic potential in the spectrum of pathological conditions, mainly because of its capacity to produce the astonishing range of proteins and matrix compartments [112].

Overall, the regulatory programs that lead to the shaping and maturation of fully functioning ChP are not extensively described with a few exceptions. For instance, the Sox9–Co9a3 axis has been recently identified as a crucial factor of the proper epithelial–basal lamina assembly whose disruption leads to the destruction of the ChPEC polarity and hyperpermeability of the blood–CSF barrier [113].

Similar polarity defects and decreased epithelial cohesivity have been observed in the HbChP of *Wnt5a* mutants [12]. Within its epithelium, Meis transcriptional factors are embroiled in the transcription of the Wnt5a from the early stages, starting at E11.5 [40]. The epithelial-specific deletion of the *Wnt5a* gene at E11.5 culminates into the severe morphological defects of the typical gross appearance of HbChP, including the decrease of its total area, reduced number of branches and their shortening [10, 40]. The severity of these malformations is further aggravated in the systemic *Wnt5a* knock-out [10, 12, 40] which can draw attention to the Wnt5a produced by stromal cells. Indeed, Dani and colleagues identified the *Wnt5a* + mesenchymal cluster [11], which suggests that Wnt5a can also mediate the crosstalk between the individual cell types fundamental for the formation of ChP structure.

Further essentiality of the fine-tuned epithelial–stroma interaction in the ChP maturation has been shown by the *Shh* knock-out mouse model which phenocopies the *Wnt5a* mutants. Here, the lack of Shh signals from the epithelium between E12.5 and E14.5 leads to the impaired function of pericytes and ultimately the reduced vascular surface area without the changes in the expression levels of typical pro-angiogenic genes like *Ang-1, Tie-2, or Vegf* [44]. Interestingly, the co-culture of endothelial cells with ChP epithelial cells, which have been observed to express *Vegf* [115], resulted in the increased numbers of ChP typical fenestrations within the endothelial cell population [116]. All these findings suggest a complex, multi-step maturation process of the ChP vasculature which involved a variety of signaling cascades needed during the different embryonic time points as the small number of fenestrated vessels have not been observed as early as E16.5 in the rat model [117].

Our understanding of the interplay between all compartments of maturating ChP can be further enhanced by recent bioinformatic analysis of the potential cell–cell interaction network across the ChP tissue. This extensive prediction, based on the ligand and receptor expression patterns within the individual cell types, highlighted mesenchymal cells (i.e., fibroblast and pericytes) as an organizing center that presumably communicates with the remaining cell types via several distinct signaling pathways including Wnt, Bmp, or Notch protein families [11].

## ChP-mediated brain patterning

Besides the blood–CSF barrier formation or the CSF secretory function, the ChP has nowadays been appreciated also as the orchestrator of the long-range signaling in the developing brain. This function of ChP is ensured by the production of numerous signaling molecules into the CSF.

The initial work demonstrating the importance of ChP induced patterning within the embryonic telencephalon comes from Lehtinen et al*.* These authors identified the presence of Igf2 within the meninges and epithelium of the TelChP, and proved that its release into the CSF culminates in the enhanced proliferation of stem cells within the ventricular–subventricular zone (V-SVZ) [8]. Further patterning of this brain region was reported in the recent study where ChP-derived Semaphorins (Sema)/Neuropilins (Nrp) complexes were identified in the CSF. Here, Gerstmann and colleagues showed a decreased rate of murine progenitor differentiation caused by the Sema3b/Nrp2 induced enhancement of their adhesion properties. In turn, Sema3F/Nrp1 complex seems to decrease cell detachment, thus inducing neuron differentiation [118]. More investigation on this issue has been performed on adult samples. For instance, *Otx2* knock-out mice display lower numbers of newborn neurons which integrate into the olfactory bulb. This is caused by the impairment of their migration properties which is connected to the alternation of extracellular matrix within the V-SVZ of animals lacking the CSF-derived Otx2 protein [9]. Furthermore, the transport of one of the most emblematic markers of ChPEC—Transthyretin—across the lateral ventricle seems to be fundamental to maintaining the balance in the differentiation of neurogenic as well as oligodendrogenic cell types within the lateroventral—SVZ progenitor niche [119]. Intriguingly, the recent study by Arnaud et al*.* also showed the interplay between TelChP and the hippocampus. Shortly, the elevated pool of App within the adult ChP leads to the impaired proliferation within hippocampus stem cells niche and behavioral defects in reversal learning [120]. Another brain region that is sensitive to the external stimuli sent out of ChP is the progenitor niche of the cerebellum. In detail, the Wnt5a protein [10] and Shh [55] have been observed to be emitted from the epithelial monolayer of HbChP and transported across the 4th ventricle to regulate cerebellar proliferation.

The secretome analysis of the media from TelChP and HbChP explants has revealed the presence of nearly 200 proteins that are likely to be produced by ChPs in vivo*.* The authors highlighted several proteins with the potential to participate in the further brain patterning (e.g., Ctsb or Ctsd in the case of TelChP or HbChP-derived Ec-sod or Penk) [54]; however, these findings need further validations in the identical model.

## Aging of the ChP and its related diseases

Severe alterations have been observed within the structure of ChP during aging. One of the first described is the so-called Biondi bodies [121]. These filamentous, lipid droplets-associated structures develop preferably in the cytoplasm of aged human ChPEC and it is speculated that these bodies have a destructive impact on the epithelial sheet [122]. Furthermore, the transcriptomic analysis of the adult and aged mouse ChP showed the expression shift related to the IL-1B signaling within the aging macrophages, endothelial, as well as mesenchymal cells. The authors ascribe this observation to enhance macrophage migration and subsequent infiltration of the CSF–blood barrier occurring in older individuals [11].

Similarly, the changes in the status or gene expression pattern of individual ChP cell types have been connected to the presence of various infectious agents in the body, for which the ChP represents the gateway to the CNS. For instance, the Zika virus disturbs the brain cortex via the ChP pericytes’ infection, impairment of the ChP epithelium, and subsequent infiltration into the CSF [123]. Single-cell analysis of human ChP tissue from COVID-19-positive patients revealed inflammatory associated transcriptional changes within the ChP epithelium [124] which is in line with the previous study performed on the latest cutting-edge model in the ChP field—human ChP organoids [41]. Here, the authors showed the leakage of the CSF–blood barrier as well as the inflammatory transition of ChPEC induced by the COVID-19 infection [125]. Intriguingly, the recent study by Carloni and colleagues uncovers the defense mechanism within the ChP tissue restricting the agens entrance and further spreading across the CNS. The authors, focusing on inflammatory bowel diseases, first detected the upregulation of the Wnt/ß-catenin signaling pathway in the ChP endothelial cells from infected animals causing the increased permeability of the ChP vascular network. To prevent further infections, the recruitment of inflammatory cells occurs resulting in the shutdown of the CSF–blood barrier [126].

Beside the entering point, ChP may also act as a reservoir of infection agents as Liu et al*.* recently showed studying the Ebola virus. Within this study, the authors showed that even after the monoclonal antibody-based treatment, the virus particles were detected within the ChP macrophage cell pool and their subsequent activity resulted in the lethal recrudescence of Ebola diseases [127].

In addition to mental disorders, which have been linked to ChP tissue dysfunction, e.g., schizophrenia [128], bipolar disorder [129], depression [130] anxiety [131] or Alzheimer's disease [132], notorious disorders of this structure are ChP carcinoma and ChP sarcoma. These ChP-based tumors are rare diseases (accounting for 2–4% of intracranial tumors in children and 0.5% in adults [133]) that are most typical for the TelChP and HbChP. Interestingly, 80% of TelChP tumors have been observed in children, while the incidence of HbChP tumors is more uniformly distributed across age groups [134]. Characteristic clinical features of ChP tumors, which have been allied to several genetic conditions, such as Aicardi syndrome [135], Li–Fraumeni syndrome [136], or Von Hippel–Lindau disease [137], are hydrocephalus and increased intracranial pressure which manifest in vomiting or headaches [138]. On the molecular level, ChP tumors have been connected to the alternation in the TP53 [139], PDGFR [140], Notch [141], or Shh signaling cascade [142].

Apart from the aforementioned syndromes, mouse models of ciliopathies such as Bardet–Biedl syndrome [143], Joubert syndrome [144], or Meckel–Gruber syndrome [145], which have already been associated with the ChP cyst [146], show cilia loss at the epithelial monolayer of the ChP. This is accompanied by the disruption of the ChPEC integrity and hydrocephalus. Altogether, these studies underline the relevance of the ChP in the research and/or diagnosis of these ciliopathies.

## Conclusion and future directions

The enormous effort has been recently put into the ChP research with the intention to fully comprehend every aspect of this secretory organ—its functions, morphogenesis, as well as mechanisms that result in the onset of ChP-related diseases. The range of models used in the ChP field is growing rapidly and includes zebrafish line with fluorescent ChPECs [63], the time- and/or tissue-specific conditional mouse knockouts [10, 55], or human ChP organoids [41]. This is accompanied by the boost of transcriptomic [11, 54, 128] and proteomic datasets [41, 54] of ChP tissue or CSF. Despite all of this, many key issues related to the ChP development, and its functions remain unresolved. First, are there any additional molecular mechanisms of ChP epithelium specification which would be shared by all ChP types? Then, is the predicted common origin of ChPs in the *Rspo2* + progenitors evolutionary conserved? What is the full extent of the epithelium–stroma interactions and what is its impact on the morphology of individual ChPs? What is the extent of regionalization of CSF within the individual ventricles? How is such diversity maintained? What is the full scope of brain regions which respond to the biomolecules produced by the ChP? Overall, further studies within the basic research framework are needed to put the entire picture of developmental processes and the functioning of this unique structure together. Only once we broaden our basic knowledge of ChP, we can understand better the onset of the ChP pathologies, and ultimately develop the effective treatments of ChP-related diseases.

## Data Availability

Not relevant.
